# 179 Physical activity and physical function in survivors of critical illness after hospital discharge: A prospective, observational study

**DOI:** 10.1093/eurpub/ckae114.179

**Published:** 2024-09-26

**Authors:** Jill Costley, Jason Wilson, Natasha Green, Judy Bradley, Danny McAuley, Bronagh Blackwood, Brenda O’neill

**Affiliations:** Centre for Health and Rehabilitation Technologies, INHR, Ulster University, Belfast, United Kingdom; Sport and Exercise Sciences Research Institute, School of Sport, Ulster University, Derry/Londonderry, United Kingdom; Wellcome-Wolfson Institute of Experimental Medicine, School of Medicine, Dentistry and Biomedical Sciences, Queen’s University Belfast, Belfast, United Kingdom; Wellcome-Wolfson Institute of Experimental Medicine, School of Medicine, Dentistry and Biomedical Sciences, Queen’s University Belfast, Belfast, United Kingdom; Wellcome-Wolfson Institute of Experimental Medicine, School of Medicine, Dentistry and Biomedical Sciences, Queen’s University Belfast, Belfast, United Kingdom; Wellcome-Wolfson Institute of Experimental Medicine, School of Medicine, Dentistry and Biomedical Sciences, Queen’s University Belfast, Belfast, United Kingdom

## Abstract

**Purpose:**

Survivors of critical illness often experience persistent physical, functional and/or cognitive impairments (i.e. Post-Intensive Care Syndrome). Currently, there is very limited rehabilitation provided as part of standard care for this population. Evaluation of physical activity (PA) and function, and the feasibility of collecting data, specifically in people after critical illness could provide insights into their activity levels after critical illness. The aim was to assess and describe PA and function in the year following hospital discharge in patients who have been mechanically ventilated in intensive care.

**Methods:**

A prospective, observational study. (Ethical approval/REC Reference: 17/N1/0115). Patients discharged from hospital following intensive care unit (ICU) admission were invited to attend up to four assessments: within 2 weeks, 6 weeks, 6 months and 1 year, following hospital discharge. Seven-day accelerometer-based PA (daily step count), modified shuttle walk test (MSWT) and handgrip strength (HGS) were assessed at each timepoint. Results were compared against research guidelines and normative data.

**Results:**

Participants (n = 14) were: age: 53.9 ± 14.5 years; sex: 8 male/6 female; ICU length of stay: 9.00 days (IQR: 4.50 days); mechanical ventilation duration: 58.50hrs (IQR: 132.75hrs); Acute Physiologic Chronic Health Evaluation: 15.36 ± 8.51. There were several challenges with data collection e.g. participants using mobility aids could not perform MSWT. Participants performed below research guidelines/normative values for mean daily steps (763 – 7744 steps), MSWT (40 – 800m), and HGS (8 – 52kg) across the 12-month study period.

**Conclusions:**

In the year following hospital discharge, PA and function varied among ICU survivors and remained low. Assessments using accelerometry and handgrip dynamometry were feasible. A more consistently administered test is needed to evaluate exercise capacity across the recovery continuum in this heterogenic population. These findings could be used to guide future development of personalised rehabilitation interventions and goal-setting in this population. Patients have also highlighted that they feel that the provision of physical rehabilitation could support their recovery.

**Support/Funding Source:**

The Department of Employment and Learning with Seed Funding from the Northern Ireland Clinical Research Facility.

